# Infidelity conduct and desire: the differentiated contribution of broad personality traits

**DOI:** 10.3389/fpsyg.2026.1939819

**Published:** 2026-08-20

**Authors:** Jose A. Rodas, Mayerly Abad, Arianna Barandica, Jose E Leon-Rojas, José Alejandro Valdevila Figueira, Daniel Oleas

**Affiliations:** 1 School of Psychology, Universidad Espíritu Santo, Samborondón, Guayas, Ecuador; 2 School of Psychology, University College Dublin, Dublin, Ireland; 3 Escuela de Medicina, Universidad de las Américas, Quito, Ecuador; 4 Escuela de Medicina, Universidad Ecotec, Samborondón, Guayas, Ecuador; 5 Department of Research, Universidad Ecotec, Samborondón, Guayas, Ecuador; 6 Department of Social Anthropology, Basic Psychology and Public Health, Universidad Pablo de Olavide, Dos Hermanas, Sevilla, Spain

**Keywords:** Big Five, Five-Factor Model, infidelity conduct, infidelity desire, intention-behavior gap, personality traits

## Abstract

**Introduction:**

Previous research linking personality to infidelity often conflates actual unfaithful conduct with the psychological desire to cheat. The present study evaluated the incremental predictive value of the Five-Factor Model (FFM) personality traits on sexual infidelity conduct and sexual infidelity desire, controlling for demographic characteristics and relationship history.

**Methods:**

A cross-sectional design was employed with a sample of 200 adults from Ecuador, who completed the Big Five Inventory and the Infidelity Conduct Subscale. Hierarchical multiple linear regression models were used to isolate the variance explained by personality dimensions.

**Results:**

The results demonstrated a clear divergence in the predictors of conduct vs. desire. Specific personality dimensions, notably higher openness to experience and lower agreeableness, significantly predicted the desire for sexual infidelity. In contrast, the predictive power of the FFM traits were not significant for actual infidelity conduct. A personal history of infidelity, alongside older age and male sex, emerged as the strongest predictors of current unfaithful conduct.

**Discussion:**

These findings provide empirical support for the intention—behavior gap within the context of romantic relationships. While personality structure generates the cognitive and emotional precursors for infidelity, actual unfaithful actions are primarily facilitated by relational history and cumulative opportunity.

## 1 Introduction

The phenomenon of romantic infidelity presents a significant threat to dyadic stability, often precipitating relationship dissolution, psychological distress, and profound interpersonal conflict (Rokach and Chan, 2023; Stavrova et al., 2022; Warach and Josephs, 2021). Understanding the individual differences that predispose individuals to extradyadic involvement is a important objective within psychology, particularly when investigating couples' satisfaction and therapeutic interventions. Personality operates as a primary psychological mechanism shaping how individuals navigate romantic commitment, temptation, and relationship dissatisfaction. Indeed, examining distinct personality configurations, such as the Dark Triad, represents a robust approach for predicting interpersonal transgressions and aversive behaviors across specific populations, including Ecuadorian adolescents (Moreta-Herrera et al., 2025, 2026). Consequently, the relationship between personality traits and extradyadic involvement has generated a substantial body of empirical research (Altgelt et al., 2018; Gewirtz-Meydan et al., 2023; Salehzadeh et al., 2024).

However, the current literature demonstrates that the predictive utility of personality is not uniform. The association between personality and infidelity varies considerably depending on the specific traits evaluated, the relational context, and crucially, the precise outcome being measured (Isma and Turnip, 2019; Salehzadeh et al., 2024; Zyl, 2021).

Within the framework of the Five-Factor model (FFM) of personality, empirical findings have identified specific traits as predictors of extradyadic involvement across cross-cultural, marital, and dating samples. The most reliable association established in the literature is the inverse relationship between conscientiousness and infidelity (Allen and Walter, 2018; Mahambrey, 2020; Schmitt, 2004). Conscientiousness encompasses self-regulation, impulse control, and reliability. Individuals exhibiting low conscientiousness report lower rates of self-reported fidelity, a higher incidence of past and lifetime infidelity, and diminished cognitive resistance when confronted with extradyadic temptation (Orzeck and Lung, 2005). This deficit in self-discipline facilitates the breach of relational boundaries, as such individuals struggle to prioritize long-term relationship preservation over immediate gratification.

Similarly, low agreeableness is recurrently linked to an increased propensity for unfaithful conduct, although the findings present slightly more heterogeneity than those for conscientiousness (Allen and Walter, 2018; Barta and Kiene, 2005; Schmitt, 2004). Agreeableness, characterized by interpersonal warmth, empathy, and cooperativeness, operates as a prosocial protective factor in romantic relationships. Individuals scoring low on this trait tend to be more self-centered and exhibit reduced empathetic concern for the potential emotional distress inflicted upon their primary partner. Consequently, low agreeableness is frequently associated with permissive attitudes toward infidelity and specific antagonistic motivations for cheating, such as seeking revenge or expressing anger toward a partner (Barta and Kiene, 2005).

Conversely, the broad traits of neuroticism, extraversion, and openness to experience yield mixed and context-dependent results. Some evidence indicates that highly neurotic individuals, prone to emotional instability and poor stress tolerance, experience chronic relationship dissatisfaction and may use extradyadic intimacy as a maladaptive strategy for emotional regulation (Allahgoliyari et al., 2026). Neuroticism has been identified as a predictor of permissive attitudes in married couples and is often linked to cheating motivated by perceived neglect or anger (Barta and Kiene, 2005; Isma and Turnip, 2019). However, the literature highlights significant inconsistencies regarding the empirical links between actual unfaithful conduct and neuroticism, extraversion, and openness. For instance, Salehzadeh et al. (2024) note that studies examining extraversion and openness have reported positive, negative, and null associations with infidelity. Furthermore, their structural equation modeling demonstrated that these personality factors influence extramarital tendencies both directly and indirectly, mediated by variables such as attachment styles and sexual satisfaction. Consequently, although extraversion theoretically increases social exposure and openness correlates with novelty-seeking, their translation into actual unfaithful behavior remains unstable across studies.

The contradictory findings surrounding these traits can be largely attributed to the profound methodological heterogeneity characterizing the field. The literature encompasses highly diverse research designs, ranging from direct comparisons between cheaters and non-cheaters to longitudinal assessments of newlyweds, cross-cultural surveys, and complex structural equation models incorporating mediating variables (Altgelt et al., 2018; Gibson et al., 2016; Orzeck and Lung, 2005; Salehzadeh et al., 2024; Schmitt, 2004). This diversity in measurement and sampling helps explain why traits like extraversion and openness frequently require the presence of additional mediating factors—such as insecure attachment styles, low sexual satisfaction, or diminished marital quality—to demonstrate a significant relationship with infidelity (Allen and Walter, 2018; Salehzadeh et al., 2024).

A critical methodological and theoretical distinction within this literature is the explicit disaggregation of actual infidelity conduct, the intention or tendency to engage in infidelity, and general attitudes or motivations toward the behavior (Isma and Turnip, 2019; Salehzadeh et al., 2024). These constructs are distinct phenomenological processes. Attitudes reflect the cognitive or moral permissiveness of the act, often predicted by traits like conscientiousness and neuroticism (Isma and Turnip, 2019; Mehrinejad and Shahabi, 2018). Motivations describe the specific psychological drivers prompting the transgression, such as dissatisfaction, neglect, anger, or the pursuit of sexual variety, which are differentially distinguished by extraversion, neuroticism, and low agreeableness (Barta and Kiene, 2005). Intention or tendency represents the conscious psychological inclination or future probability of crossing relational boundaries, which can be predicted by all five major traits depending on the sample characteristics (Salehzadeh et al., 2024). Actual conduct, conversely, represents the historical or current actualisation of these transgressions (Orzeck and Lung, 2005).

The distinction between these outcomes is central to understanding the etiology of unfaithful behavior. It is plausible that personality structure is primarily indirectly related to actual cheating behavior. Rather than functioning as a direct, deterministic trigger for unfaithful actions, personality traits might instead shape the cognitive and emotional precursors for infidelity. For instance, traits such as high openness to experience or low agreeableness could theoretically establish a psychological environment conducive to the desire, intention, or tendency to cheat, dictating an individual's attitudinal readiness and psychological inclination toward extradyadic intimacy. Under this assumption, the translation of this psychological intention into actual unfaithful behavior would represent a distinct conceptual step and require a specific personality profile and environmental facilitators.

Consequently, the transition from intention to conduct may require the alignment of proximate situational variables and could be heavily mediated by an individual's relational background. It is possible that an individual possesses a personality profile that predisposes them to desire extradyadic intimacy, yet they never engage in unfaithful conduct due to strict partner monitoring, high relationship commitment, or a simple lack of opportunity. Conversely, actual unfaithful conduct might require situational affordances, such as the availability of attractive alternatives, specific socio-cultural scripts, and a permissive relational history (Gibson et al., 2016). Therefore, it can be hypothesized that personality acts as a foundational factor generating infidelity intentions, which may ultimately lead to cheating behavior only when external and historical conditions permit. Advancing the understanding of infidelity thus requires examining how personality traits independently contribute to both the desire and the act, while isolating the influence of relational history and demographic variables.

The present study aims to evaluate the incremental predictive value of the Big Five personality traits on sexual infidelity conduct and sexual infidelity desire, beyond the effects of demographic characteristics and relationship history. A secondary aim is to determine the extent to which an individual's relational background, specifically previous relationship count and personal or partner experiences of infidelity, accounts for these behavioral and intentional dimensions. Although the theoretical rationale is compatible with a mediation framework wherein personality influences desire, which subsequently leads to conduct, a hierarchical multiple regression approach was selected. This analytical strategy isolates the variance accounted for by historical and demographic covariates, establishing the distinct, direct predictive utility of personality traits on conduct vs. desire. Given the cross-sectional design, establishing these independent associations represents a necessary foundational step before testing directional pathways in future mediation models.

## 2 Materials and methods

### 2.1 Participants

A cross-sectional design was employed for this study. The sample comprised 200 adults residing in Guayaquil and Samborondón, Ecuador, aged between 18 and 56 years (*M* = 26.66, SD = 7.77). Participants were selected using non-probabilistic convenience sampling. Inclusion criteria required participants to have current or past experience in a monogamous romantic relationship, to provide informed consent, and to demonstrate adequate comprehension of the administered instruments. Individuals who failed to complete all questionnaires were excluded. The final sample consisted of 126 females (63%) and 74 males (37%).

Sample size was determined based on a power analysis evaluating the statistical power of the hierarchical multiple regression analyses, specifically regarding the addition of the five personality dimensions in the second step. Assuming an alpha level of 0.05 and an *F*-test for the change in *R*^2^ with five tested predictors and five covariates, 200 participants provides a statistical power of 0.99 to detect a medium effect size (*f*^2^ =0.15; Cohen, 1988). Based on the actual effect sizes observed in the study, the model predicting sexual infidelity desire (*f*^2^ =0.066) achieved an adequate statistical power of 0.80.

### 2.2 Instruments

#### 2.2.1 Demographic questionnaire

An *ad hoc* questionnaire was used to collect contextual and demographic information. The instrument gathered data regarding age, sex, sexual orientation, marital status, current relationship status, relationship duration, number of previous romantic relationships, and personal or partner history of infidelity. Sex was evaluated rather than gender identity to account for the biological and social impacts on participant development. Sexual orientation response categories included heterosexual, bisexual, and prefer not to say. The number of previous romantic relationships was self-defined by participants without specific duration or formality criteria. Finally, personal and partner history of infidelity were assessed using direct dichotomous questions (i.e., “Have you been unfaithful?” and “Have you been cheated on?”, respectively).

#### 2.2.2 Multidimensional inventory of infidelity—infidelity tendency subscale

The Infidelity Tendency Subscale of the Multidimensional Inventory of Infidelity (IMIN; García et al., 2017; Romero-Palencia et al., 2007) was used to assess infidelity behaviors and intentions. The 26-item instrument is organized into four factors: sexual infidelity, emotional infidelity, desire for sexual infidelity, and desire for emotional infidelity. Items are rated on a five-point Likert scale ranging from one (never) to five (always). Higher scores indicate a greater presence of infidelity conduct or desire. The subscale has demonstrated high internal consistency (α = 0.98) and adequate construct validity (García et al., 2017). For the present study, only sexual infidelity was investigated. This decision was made to maintain conceptual clarity and analytical focus, as sexual and emotional infidelity are often driven by distinct psychological mechanisms and social scripts. Consequently, combining them could obscure trait-specific associations, meaning that emotional infidelity warrants a separate, dedicated investigation.

#### 2.2.3 Big Five Inventory

Personality traits were evaluated using the Spanish adaptation of the BFI (Benet-Martínez and John, 1998; John et al., 1991), which assesses the core dimensions of the Five-Factor Model originally operationalised by Costa and McCrae (1992). This adaptation constitutes a culturally validated instrument rather than a mere literal translation, distinguishing it from other translated versions (e.g., Silva et al., 1994). The 44-item questionnaire assesses five dimensions of personality: openness to experience, conscientiousness, extraversion, agreeableness, and neuroticism. Responses are recorded on a five-point Likert scale ranging from one (strongly disagree) to five (strongly agree). Scores for each dimension are obtained by summing the respective items, adjusting for reverse-scored items, with higher scores reflecting a greater presence of the trait. The Spanish version presents adequate convergent and discriminant validity, with internal consistencies ranging from 0.73 to 0.80 across the factors (Benet-Martínez and John, 1998).

### 2.3 Procedure

Data collection was conducted entirely online using Google Forms. The sample was recruited through three channels: digital distribution of the survey link at two academic institutions, physical flyers distributed in public spaces, and study advertisements on social media. Prior to accessing the questionnaires, participants were presented with an online informed consent document detailing the study's objectives, the voluntary nature of participation, the confidentiality of the data, and its strictly academic purpose. Only individuals who explicitly provided consent were permitted to proceed.

The study design were reviewed and formally approved by the Institutional Review Board Chair for the Psychology Program at Universidad Ecotec (Certificate No. 23-01-12-2025). All procedures were conducted in strict accordance with the ethical standards of the institutional research committee and the 1964 Declaration of Helsinki and its subsequent amendments. Prior to participation, all individuals were fully informed about the study objectives, the voluntary nature of their participation, and their right to withdraw at any stage without penalty. All participants subsequently provided online informed consent. To guarantee participant confidentiality, all data were completely anonymized prior to analysis, and personal identifying information was stored separately from the psychometric survey responses.

All measures administered during the data collection process are reported in this manuscript; no additional psychometric instruments were included in the assessment.

### 2.4 Data Analyses

Data analyses were conducted using R (R Core Team, 2026), the lavaan package (Rosseel et al., 2025), and psych package (Revelle, 2026). Descriptive statistics were computed to characterize the sample and assess the distribution of the study variables. To examine the predictive value of demographic variables, relationship history, and personality traits on infidelity conduct and desire, hierarchical multiple linear regression analyses were performed. Categorical predictors, such as sex and history of infidelity, were dummy coded prior to analysis. For each infidelity outcome, demographic and relational history variables were entered in the first step to establish a baseline model, while the five personality dimensions were entered in the second step to evaluate their incremental predictive utility over the covariates.

## 3 Results

### 3.1 Descriptive statistics

Table 1 presents the descriptive statistics for the assessed personality traits and infidelity dimensions, detailing the means and standard deviations for the overall sample and by sex. Participants reported low to moderate levels across both infidelity dimensions. Males exhibited higher mean scores in both conduct and desire for sexual infidelity compared to females. Mean scores for the FFM personality traits were consistent between sexes, with openness to experience and agreeableness presenting the highest values in the overall sample.

**Table 1:** Descriptive statistics for the study variables by sex and overall sample.

Variable	Overall (*N* = 200)	Female (*n* = 126)	Male (*n* = 74)
Sexual infidelity conduct	8.61 (3.73)	7.44 (1.44)	10.59 (5.30)
Sexual infidelity desire	9.13 (4.55)	7.90 (3.00)	11.22 (5.83)
Extraversion	24.52 (3.66)	24.62 (3.71)	24.34 (3.60)
Neuroticism	23.86 (4.24)	24.12 (4.31)	23.43 (4.13)
Openness to experience	29.94 (8.00)	30.33 (7.44)	29.28 (8.90)
Conscientiousness	27.77 (3.25)	27.84 (3.43)	27.65 (2.96)
Agreeableness	30.42 (4.99)	30.81 (5.34)	29.76 (4.29)

Data are presented as M (SD).

### 3.2 Hierarchical Regression Analyses

Two hierarchical multiple linear regression analyses were conducted to evaluate the predictive value of demographic variables, relationship history, and personality traits on sexual infidelity conduct and sexual infidelity desire. For both models, age, sex, number of previous relationships, personal history of infidelity, and partner history of infidelity were entered in the first block. The five personality dimensions were entered in the second block. Results from these models are presented in Table 2.

**Table 2:** Hierarchical regression analysis predicting sexual infidelity conduct and desire.

Predictor	Conduct			Desire		
	*B*	SE	β	*B*	SE	β
Step 1						
Age	0.06^**^	0.02	0.13	0.00	0.03	0.01
Sex	2.08^***^	0.38	0.27	2.13^***^	0.53	0.23
Previous relationships	0.25^*^	0.12	0.12	0.56^***^	0.16	0.22
Personal history	4.82^***^	0.50	0.54	4.53^***^	0.69	0.42
Partner history	−0.15	0.37	−0.02	−0.18	0.52	−0.02
Step 2						
Age	0.07^**^	0.02	0.15	0.02	0.03	0.04
Sex	2.09^***^	0.38	0.27	2.08^***^	0.53	0.22
Previous relationships	0.22	0.12	0.11	0.49^**^	0.16	0.19
Personal history	4.95^***^	0.51	0.55	4.76^***^	0.70	0.44
Partner history	−0.18	0.38	−0.02	−0.23	0.52	−0.03
Extraversion	−0.04	0.06	−0.04	−0.06	0.08	−0.05
Neuroticism	0.01	0.05	0.01	0.04	0.07	0.03
Openness	0.06	0.03	0.13	0.13^**^	0.05	0.23
Conscientiousness	0.01	0.07	0.00	0.04	0.09	0.03
Agreeableness	−0.04	0.05	−0.05	−0.18^*^	0.07	−0.20

Sex is coded 0 = female, 1 = male. Personal history and partner history are coded 0 = no, 1 = yes. B, unstandardized regression coefficients; SE, standard errors; β, standardized regression coefficients.

^*^p < 0.05, ^**^p < 0.01, ^***^p < 0.001.

The first block of predictors accounted for 55.9% of the variance in sexual infidelity conduct, *R*^2^ = 0.559, *F*_(5, 194)_ = 49.08, *p* < 0.001. Age, sex, previous relationships, and personal history of infidelity were significant predictors. The introduction of the personality traits in the second block did not significantly improve the model, Δ*R*^2^ = 0.009, *F*_(5, 189)_ = 0.83, *p* = 0.533. In the final model, age (β = 0.15), sex (β = 0.27), and a personal history of infidelity (β = 0.55) remained significant predictors. The personality variables were not significantly associated with actual infidelity behavior.

The first block explained 41.6% of the variance in the desire for sexual infidelity, *R*^2^ =0.416, *F*_(5, 194)_ = 27.65, *p* < 0.001, with sex, previous relationships, and personal history of infidelity emerging as significant predictors. Adding the personality traits in the second block yielded a significant improvement in the model, Δ*R*^2^ = 0.036, *F*_(5, 189)_ = 2.48, *p* = 0.033. The final model accounted for 45.2% of the variance, *R*^2^ = 0.452, *F*_(10, 189)_ = 15.59, *p* < 0.001. Alongside sex (β = 0.22), previous relationships (β = 0.19), and personal history of infidelity (β = 0.44), two personality traits significantly predicted infidelity desire: openness to experience showed a positive association (β = 0.23), whereas agreeableness demonstrated a negative association (β = −0.20).

## 4 Discussion

The current study aimed to evaluate the predictive role of the FFM personality traits on sexual infidelity conduct and desire, controlling for demographic variables and relationship history. The findings indicate that actual infidelity conduct is primarily predicted by demographic and historical factors—specifically older age, male sex, and a personal history of infidelity—with personality traits offering no additional predictive value. Conversely, personality significantly predicts the desire for sexual infidelity. Alongside male sex, a higher number of previous relationships, and a personal history of infidelity, higher openness to experience and lower agreeableness emerged as significant predictors of infidelity desire. These results indicate that while broad personality traits shape the inclination toward infidelity, actual cheating behavior is more strongly dictated by past relational conduct and demographic variables.

The absence of significant associations between the FFM personality traits and sexual infidelity conduct contradicts some previous literature that often links low conscientiousness and high extraversion or neuroticism to unfaithful behavior (Allen and Walter, 2018; Mahambrey, 2020; Salehzadeh et al., 2024). However, this discrepancy highlights the importance of the hierarchical analytical approach used in this study, since demographic and past behavior significantly contribute to the model. A personal history of infidelity emerged as the strongest predictor of current infidelity conduct, supporting the behavioral consistency premise that past behavior is the most reliable predictor of future behavior (Orzeck and Lung, 2005). Individuals who have crossed the boundary of exclusivity in the past may experience a reduction in cognitive dissonance or guilt, making subsequent unfaithful acts more permissible. Furthermore, the significant predictive role of older age and male sex suggests that actual infidelity conduct is heavily influenced by cumulative opportunity and socio-cultural scripts. Older individuals have simply had more time and exposure to situations where infidelity could occur, while traditional gender socialization often affords males greater leniency or even implicit encouragement regarding sexual permissiveness. According to sexual script theory (Simon and Gagnon, 1986, 2003), men are socialized to associate masculine competence with sexual pursuit and high levels of desire. Furthermore, masculinity theory suggests that turning down sexual opportunities is stigmatized, placing social pressure on men to project constant sexual readiness (Masters et al., 2013), which can result in outward sexual performance diverging from internal physical drive (Murray, 2018). Conversely, women are frequently stigmatized for similar behaviors and must often unpack embedded gender inequalities to endorse sexual permissiveness (Ciaffoni et al., 2025; Conley and Klein, 2022). While evolutionary psychology posits that males may exhibit a higher propensity for sexual variety as a mating strategy (Barta and Kiene, 2005; Schmitt, 2004), these sociocultural constructions and traditional masculine norms provide a robust explanatory framework for the persistent effect of sex on both conduct and desire across the regression models.

Consequently, actual infidelity appears less as a direct manifestation of a specific personality profile and more as a repeated behavioral pattern facilitated by demographic, historical opportunities and individual characteristics (i.e., personality).

In contrast to actual conduct, the desire for sexual infidelity was significantly predicted by specific personality dimensions, demonstrating that the psychological inclination toward extradyadic intimacy operates under different mechanisms than the behavior itself (Isma and Turnip, 2019; Zyl, 2021). Higher openness to experience significantly predicted infidelity desire. Openness is characterized by a preference for novelty, variety, and a willingness to challenge conventional social norms (Salehzadeh et al., 2024). In the context of romantic relationships, highly open individuals may experience greater curiosity about alternative partners and a stronger aversion to the routine that often accompanies long-term monogamy. This trait does not necessarily compel them to act unfaithfully, but it creates a fertile cognitive environment for fantasizing about or desiring extradyadic sexual encounters.

Similarly, lower agreeableness emerged as a significant predictor of the desire for sexual infidelity. Agreeableness encompasses interpersonal warmth, empathy, altruism, and cooperation. Individuals with lower levels of this trait tend to be more self-centered, competitive, and less sensitive to the emotional needs or potential suffering of others. In romantic relationships, low agreeableness may translate into a diminished sense of commitment and a prioritization of personal sexual gratification over the relational bond (Barta and Kiene, 2005). When the desire for alternative sexual experiences arises, individuals low in agreeableness may feel fewer internal moral constraints or empathetic concern for their primary partner's potential distress, allowing the desire to persist and strengthen (Allen and Walter, 2018). The finding that agreeableness predicts the intention but not the action suggests that while disagreeable individuals may readily entertain the idea of cheating, external factors or practical constraints must still align for the behavior to manifest.

The divergence in predictors for infidelity conduct vs. desire provides empirical support for the intention-behavior gap within the context of romantic relationships. While personality traits such as high openness and low agreeableness generate the cognitive and emotional precursors for infidelity, translating these desires into action requires specific situational affordances (Gibson et al., 2016). A person may strongly desire an extradyadic encounter due to their personality structure but refrain from acting out of fear of discovery, lack of a willing alternative partner, or strict partner monitoring. Conversely, an individual who does not inherently desire infidelity might still engage in unfaithful conduct if placed in a highly facilitating environment, especially if they possess a history of similar behavior that has desensitized them to the consequences. This distinction is crucial for understanding the complex etiology of infidelity. It suggests that researchers must move beyond attempting to identify a singular infidelity personality profile and instead examine how personality traits interact with relational history and situational opportunities to either suppress or facilitate unfaithful actions (Altgelt et al., 2018; Gibson et al., 2016).

These findings offer explicit theoretical and practical implications. Theoretically, the study reinforces the necessity of disaggregating psychological desire from behavioral enactment in infidelity research, providing evidence that broad personality traits primarily inform the former. Practically, these results can inform clinical assessments and therapeutic interventions in relationship counseling. Clinicians should recognize that while a partner's personality profile, such as high openness or low agreeableness, may necessitate interventions focused on managing extradyadic desires and communicating relational boundaries, actual behavioral risk is more accurately assessed through a thorough evaluation of the individual's relational history and demographic context. Therapeutic strategies aiming to prevent or address extradyadic involvement may therefore benefit from targeting historical behavioral patterns and situational opportunities rather than focusing solely on individual personality traits.

## 5 Limitations and future directions

Despite the methodological strengths of the present study, several limitations must be acknowledged. First, the cross-sectional design precludes the establishment of causal relationships. While it is theoretically sound to position personality and past behavior as predictors of current conduct and desire, longitudinal research is required to track how these variables interact and influence relationship trajectories over time. Second, the reliance on self-report measures introduces the risk of social desirability bias. Although the data collection was anonymous, the sensitive nature of infidelity may lead to underreporting, particularly regarding actual unfaithful conduct. Finally, the sample was obtained through non-probabilistic convenience sampling in Ecuador and primarily consisted of young adults. This restricts the generalizability of the findings to different cultural contexts or older populations where relational dynamics and attitudes toward infidelity might differ significantly. Furthermore, the relational framework underpinning this study implicitly relies on monogamous assumptions. Infidelity is likely experienced and conceptualized differently within consensual non-monogamous (CNM) relationships. Consequently, future research would benefit from integrating more diverse demographic samples and explicitly examining these constructs across varying relationship structures to delimit the scope of these conclusions. Additionally, given our finding that personality does not directly predict infidelity behavior, future studies should use mediation models to examine how personality traits influence infidelity desire as an intermediary step toward conduct.

Furthermore, this study assessed broad personality domains at the individual level, which does not account for the dyadic nature of romantic relationships or more granular trait facets. Contemporary research using dyadic samples underscores the premise that personality traits do not operate in a vacuum; a partner's characteristics and couple-level processes dynamically interact to either buffer against or precipitate extradyadic behavior (Altgelt et al., 2018). Future research should explicitly account for the dyadic nature of relationships to evaluate how partner characteristics interact with individual traits. Likewise, investigating specific personality facets may clarify the psychological mechanisms driving infidelity that broad domains obscure (Zyl, 2021).

## Data Availability

The raw data supporting the conclusions of this article will be made available by the authors, without undue reservation.

## References

[B1] Allahgoliyari M. Ajdarbin B. Aghmiuni S. R. (2026). Prediction of infidelity tendency based on love styles and personality traits in married students. J. Adolesc. Youth Psychol. Stud. 7, 1–13. doi: 10.61838/kman.jayps.4894

[B2] Allen M. Walter E. (2018). Linking Big Five personality traits to sexuality and sexual health: a meta-analytic review. Psychol. Bull. 144, 1081–1110. doi: 10.1037/bul0000157 29878796

[B3] Altgelt E. E. Reyes M. A. French J. E. Meltzer A. L. McNulty J. K. (2018). Who is sexually faithful? Own and partner personality traits as predictors of infidelity. J. Soc. Pers. Relatsh. 35, 600–614. doi: 10.1177/0265407517743085

[B4] Barta W. D. Kiene S. M. (2005). Motivations for infidelity in heterosexual dating couples: the roles of gender, personality differences, and sociosexual orientation. J. Soc. Pers. Relatsh. 22, 339–360. doi: 10.1177/0265407505052440

[B5] Benet-Martínez V. John O. P. (1998). Los cinco grandes: across cultures and ethnic groups: multitrait-multimethod analyses of the Big Five in Spanish and English. J. Pers. Soc. Psychol. 75, 729–740. 9781409 10.1037//0022-3514.75.3.729

[B6] Ciaffoni S. Rubini M. Moscatelli S. (2025). The unbearable weight of gender inequalities: development and validation of the Social Treatment and Experiences of Women (STEW) scale. Sex Roles 91, 1–18. doi: 10.1007/s11199-024-01555-140207095

[B7] Cohen J. (1988). Statistical Power Analysis for the Behavioral Sciences, 2nd Edn. Mahwah, NJ: Lawrence Erlbaum Associates.

[B8] Conley T. D. Klein V. (2022). Women get worse sex: a confound in the explanation of gender differences in sexuality. Perspect. Psychol. Sci. 17, 960–978. doi: 10.1177/17456916211041598 35171743

[B9] Costa P. T. McCrae R. R. (1992). Revised NEO Personality Inventory (NEO PI-R) and NEO Five-Factor Inventory (NEO-FFI) Professional Manual. Odessa, FL: Psychological Assessment Resources.

[B10] García M. A. Rivera S. Ramírez G. (2017). Validación del Inventario Multidimensional de Infidelidad (IMIN) en una muestra mexicana. Rev. Iberoam. Diagn. Eval. Psicol. 2, 163–175.

[B11] Gewirtz-Meydan A. Mitchell K. J. O'Brien J. E. (2023). Sexual posttraumatic stress among investigators of child sexual abuse material. Policing 17:paad052. doi: 10.1093/police/paad052

[B12] Gibson K. Thompson A. E. O'Sullivan L. F. (2016). Love thy neighbour: Personality traits, relationship quality, and attraction to others as predictors of infidelity among young adults. Can. J. Hum. Sex. 25, 186–198. doi: 10.3138/cjhs.253-a2

[B13] Isma M. N. P. Turnip S. S. (2019). Personality traits and marital satisfaction in predicting couples' attitudes toward infidelity. J. Relatsh. Res. 10:e14. doi: 10.1017/jrr.2019.10

[B14] John O. P. Donahue E. M. Kentle R. L. (1991). The Big Five Inventory–Versions 4a and 54. Berkeley, CA: Institute of Personality and Social Research, University of California, Berkeley.

[B15] Mahambrey S. R. (2020). Self-reported Big Five personality traits of individuals who have experienced partner infidelity. Pers. Relatsh. 27, 274–302. doi: 10.1111/pere.12315

[B16] Masters N. T. Casey E. Wells E. A. Morrison D. M. (2013). Sexual scripts among young heterosexually active men and women: continuity and change. J. Sex Res. 50, 409–420. doi: 10.1080/00224499.2012.661102 22489683 PMC3515716

[B17] Mehrinejad A. Shahabi F. (2018). Prediction of attitude toward extramarital relationships based on impulsivity and personality traits. J. Res. Health: 8, 492–500. doi: 10.29252/jrh.8.6.492

[B18] Moreta-Herrera R. Larzabal-Fernández A. Rodas J. A. Rodríguez-Lorenzana A. Pilco K. (2025). Psychometric properties of the 12 items Dark Triad of Personality Scale (Dirty Dozen Scale) in Ecuadorian adolescents: analysis from the classical test and item response theory. Rev. Psicol. Clín. Niños Adolesc. 12, 168–177. doi: 10.21134/RPCNA.2025.12.3.3

[B19] Moreta-Herrera R. Núñez-Núñez M. Rodas J. A. Paredes-Proaño A. M. Oriol-Granado X. (2026). Dark triad personality traits, bullying and cyberbullying among adolescents in Ecuador: a psychological network analysis. J. Aggress: Conflict Peace Res. 1–18. doi: 10.1108/JACPR-01-2026-1103

[B20] Murray S. H. (2018) Heterosexual men's sexual desire: supported by, or deviating from, traditional masculinity, norms, and sexual scripts? Sex Roles 78, 130–141. doi: 10.1007/s11199-017-0766-7.

[B21] Orzeck T. Lung E. (2005). Big-five personality differences of cheaters and non-cheaters. Cur. Psych. 24, 274–286. doi: 10.1007/s12144-005-1028-3

[B22] R Core Team (2026). R: A Language and Environment for Statistical Computing (Version 4.4.x) [Computer software]. Vienna: R Foundation for Statistical Computing. doi: 10.32614/R.manuals

[B23] Revelle W. (2026). psych: Procedures for Psychological, Psychometric, and Personality Research (Version 2.6.3) [Computer Software]. Northwestern University. Aailable online at: https://CRAN.R-project.org/package=psych (Accessed July 14, 2026).

[B24] Rokach A. Chan S. (2023). Love and infidelity: causes and consequences. Int. J. Environ. Res. Public Health 20. doi: 10.3390/ijerph20053904 36900915 PMC10002055

[B25] Romero-Palencia A. Rivera-Aragón S. Díaz-Loving R. (2007). Desarrollo del inventario multidimensional de infidelidad (IMIN). Rev. Iberoam. Diagn. Eval. Psicol. 23, 121–148. Available online at: https://aidep-aidap.org/revista/0023/RIDEP023-Art07.pdf (Accessed August 11, 2026).

[B26] Rosseel Y. Jorgensen T. D. De Wilde L. (2025). lavaan: Latent Variable Analysis (Version 0.6-20) [Computer software]. Ghent: Comprehensive R Archive Network. doi: 10.32614/CRAN.package.lavaan

[B27] Salehzadeh S. Abadi B. A. G. H. Nejat H. (2024). Structural equation modeling of the tendency toward marital infidelity based on significant five personality factors mediated by attachment styles and sexual satisfaction. J. Health Rep. Technol. e142998. doi: 10.5812/jhrt-142998

[B28] Schmitt D. P. (2004). The Big Five related to risky sexual behaviour across 10 world regions: differential personality associations of sexual promiscuity and relationship infidelity. Eur. J. Pers. 18, 301–319. doi: 10.1002/per.520

[B29] Silva F. Avia M. D. Sanz J. Martínez-Arias R. Graña J. L. Sánchez-Bernardos M. L. . (1994). NEO PI-R. Inventario de Personalidad NEO Revisado, y NEO-FFI. Inventario NEO Reducido de Cinco Factores. Madrid: TEA Ediciones.

[B30] Simon W. Gagnon J. H. (1986). Sexual scripts: permanence and change. Arch. Sex. Behav. 15, 97–120. doi: 10.1007/BF01542219 3718206

[B31] Simon W. Gagnon J. H. (2003). Sexual scripts: origins and development. Qual. Sociol. 26, 491–497. doi: 10.1023/B:QUAS.0000005053.99846.e5

[B32] Stavrova O. Pronk T. M. Denissen J. J. A. (2022). Estranged and unhappy? Examining the dynamics of personal and relationship WellBeing Surrounding Infidelity. Psychol. Sci. 34, 143–169. doi: 10.1177/09567976221116892 36322915

[B33] Warach B. Josephs L. (2021). The aftershocks of infidelity: a review of infidelity-based attachment trauma. Sex. Relatsh. Ther. 36, 68–90. doi: 10.1080/14681994.2019.1577961

[B34] Zyl C. J. (2021). The five factor model and infidelity: beyond the broad domains. Pers. Individ. Dif : 172:110553. doi: 10.1016/j.paid.2020.110553

